# Effects of a weight loss supplement on body composition and markers of metabolism in overweight male and female adults

**DOI:** 10.1186/1550-2783-10-S1-P27

**Published:** 2013-12-06

**Authors:** Jordan Outlaw, Sara Hayward, Stacie Urbina, Josh Holt, Brooke Cox, Bailey Burks, Eliza Faillace, Matthew Stone, Brittany Stai, Cliffa Foster, Chris Lockwood, Mike Roberts, Colin Wilborn, Lem Taylor

**Affiliations:** 1 Department of Exercise & Sports Science, Human Performance Lab, University of Mary Hardin-Baylor, Belton, TX 76531, USA; 2 Department of Exercise & Sports Science, Exercise Biochemistry Lab, University of Mary Hardin-Baylor, Belton, TX 76531, USA; 3 4Life Research, Sandy, UT 84070, USA; 4 Department of Biomedical Sciences, Booth Laboratory, University of Missouri-Columbia, Columbia, MO 65211, USA

## Background

Various weight loss supplements are commercially available and are composed of a wide variety of ingredients. Combined with a low calorie diet, some dietary supplements could possibly lead to changes in metabolism and/or suppression of appetite that could lead to improved body composition. The purpose of this study was to investigate the effects of ingesting a commercially available dietary supplement and its effects on body composition, resting energy expenditure (REE), hunger, and various blood markers in free-living, overweight individuals.

## Methods

Fifty-four male and female (40.7 ± 8.28 yrs, 90.82 ± 15.62 kg, 34.02 ± 7.42 %BF) subjects completed both acute (2.5 hours) and sub-acute (8 days) testing in a double-blind and placebo controlled design. Participants were divided into three groups: placebo (PL), high dose (EXP1), and standard dose (EXP2) in a matched-pair, randomized manner based on %BF. Baseline measurements included body composition via DEXA, blood collection, hunger scale, hemodynamics, and REE. Participants consumed the supplement and repeated testing at various time points for a period of 2 hours while resting in a supine position. Participants consumed the supplement (proprietary blend of: L-arginine, L-carnitine, L-ornithine, EGCG, saffron extract, black cohosh) for 7 days (daily dose per group: EXP1: 3032 mg; EXP2: 1516 mg) and repeated all testing. Dependent variables were analyzed as means and delta (Δ) responses from baseline using a 2-way (group X time) ANOVA with repeated measures (p < 0.05).

## Results

Significant main effect for time was seen for Δfat mass (*p* = 0.002), Δbody mass (*p* = 0.029), and Δ%BF (*p* = 0.006). A trend for significance (p = 0.08) was observed for %BF, indicating a possible benefit for a reduction in body fat in the standard dose group (EXP2). Change in %BF from baseline was greatest in EXP2 (PL: -0.167 ± 1.17, EXP1: -0.23 ± 0.93, EXP2: -1.01 ± 1.49 Δ%BF). Significant main effect for time (*p* = 0.000) and a group x time interaction for acute free fatty acid (FFA) appearance (T1: *p* = 0.000; T2: *p* = 0.014) were observed. Post-hoc testing indicated FFA levels rose significantly at 90 and 120 mins in EXP2, while PL significantly decreased over the same time period. Despite mean increases in REE, no differences for time or group were observed. No negative effects on blood (complete metabolic panel/CBC) or hemodynamic (SBP, DBP, RHR) safety variables were observed.

## Conclusion

Results of this study indicate that ingestion of a commercially available weight loss supplement significantly increased FFA levels in the blood in an acute fashion. Considering the short supplementation period (8 days), the observed trends for changes in percent body fat and fat mass suggest a possible benefit on body composition when consuming the regularly suggested dose. Longer periods of supplementation may have a possible significant effect on those body composition measures. Ingestion of this dietary supplement is apparently safe (blood/hemodynamic measures) in an acute and a sub-acute setting.

